# Study of a Lead-Free Perovskite Solar Cell Using CZTS as HTL to Achieve a 20% PCE by SCAPS-1D Simulation

**DOI:** 10.3390/mi12121508

**Published:** 2021-12-01

**Authors:** Ana C. Piñón Reyes, Roberto C. Ambrosio Lázaro, Karim Monfil Leyva, José A. Luna López, Javier Flores Méndez, Aurelio H. Heredia Jiménez, Ana L. Muñoz Zurita, Francisco Severiano Carrillo, Esteban Ojeda Durán

**Affiliations:** 1 Centro de Investigaciones en Dispositivos Semiconductores (CIDS-ICUAP), Benemérita Universidad Autónoma de Puebla (BUAP), Av. San Claudio y 14 sur, Edif. IC5 C.U., Col. San Manuel, Puebla C.P. 72570, Mexico; karim.monfil@correo.buap.mx (K.M.L.); jose.luna@correo.buap.mx (J.A.L.L.); esteban.ojeda@alumno.buap.mx (E.O.D.); 2 Facultad de Electrónica, Benemérita Universidad Autónoma de Puebla (BUAP)-Ciudad Universitaria, Blvd. Valsequillo y Esquina, Av. San Claudio s/n, Col. San Manuel, Puebla C.P. 72570, Mexico; javier.floresme@correo.buap.mx (J.F.M.); analuz.munoz@correo.buap.mx (A.L.M.Z.); 3 Facultad de Electrónica, UPAEP, 21 sur No. 1103, Barrio de Santiago, Puebla C.P. 72410, Mexico; aureliohoracio.heredia@upaep.mx; 4 Instituto Politécnico Nacional, Centro de Investigación en Biotecnología Aplicada Unidad Tlaxcala, Carretera a Santa Inés Tecuexcomac, a 1.5 km, Ex-Hacienda San Juan Molino, Tlaxcala C.P. 90700, Mexico; fseveriano@conacyt.mx

**Keywords:** CZTS, MASnI_3_, solar cell, SCAPS-1D, HTL, ETL

## Abstract

In this paper, a *n-i-p* planar heterojunction simulation of Sn-based iodide perovskite solar cell (*PSC*) is proposed. The solar cell structure consists of a Fluorine-doped tin oxide (FTO) substrate on which titanium oxide (TiO_2_) is placed; this material will act as an electron transporting layer (ETL); then, we have the tin perovskite CH_3_NH_3_SnI_3_ (MASnI_3_) which is the absorber layer and next a copper zinc and tin sulfide (CZTS) that will have the function of a hole transporting layer (HTL). This material is used due to its simple synthesis process and band tuning, in addition to presenting good electrical properties and stability; it is also a low-cost and non-toxic inorganic material. Finally, gold (Au) is placed as a back contact. The lead-free perovskite solar cell was simulated using a Solar Cell Capacitance Simulator (SCAPS-1D). The simulations were performed under AM 1.5G light illumination and focused on getting the best efficiency of the solar cell proposed. The thickness of MASnI_3_ and CZTS, band gap of CZTS, operating temperature in the range between 250 K and 350 K, acceptor concentration and defect density of absorber layer were the parameters optimized in the solar cell device. The simulation results indicate that absorber thicknesses of 500 nm and 300 nm for CZTS are appropriate for the solar cell. Further, when optimum values of the acceptor density (*N_A_*) and defect density (*N_t_*), 10^16^ cm^−3^ and 10^14^ cm^−3^, respectively, were used, the best electrical values were obtained: *J_sc_* of 31.66 mA/cm^2^, *V_oc_* of 0.96 V, *FF* of 67% and *PCE* of 20.28%. Due to the enhanced performance parameters, the structure of the device could be used in applications for a solar energy harvesting system.

## 1. Introduction

Energy harvesting (EH) has become a research topic due to the scavenging of ambient energy and its conversion into electrical energy for charging batteries and therefore powering electronic devices. Some useful external sources are solar power, thermal, Radio Frequency (RF), mechanical and vibrations which can report power in the range from nW to mW [1]. In particular, the use of solar energy harvesting technology has increased enormously around the world [2]. Solar energy harvesting requires efficient and low-cost photovoltaic devices, but they also need to be environmentally friendly. This is one of the reasons for developing solar cells with new materials that could increase the efficiency [3] and avoid negative effects on the environment.

A great number of theorical and experimental investigations have been carried out to improve the performance of the power conversion efficiency (*PCE*) in perovskite solar cells (*PSC*). C, Devi et al. [4] studied lead perovskite-based solar cells such as a MAPbX_3_ (X = I, Cl, Br) structure, achieving up to 24.10%. T. Fujihara, S. et al. [5] developed an appropriate formation process for tin perovskite films; they obtained flat perovskite layers with a high surface coverage and a *PCE* of about 2.14 ± 0.35%.

Studies such as Ying-Chiao Wang et al. [6] have proposed an ETL-assisted nucleation and a fabrication process of planar heterojunction *n-i-p* structured *PSCs* with *PCE* values greater than 20%. Ying-Chiao Wang et al. [7] introduced silicon quantum dots (SiQDs) into *PSCs* to achieve a *PCE* enhancement from 18.2% up to 19.6%.

Faisal Baig et al. [8] and Intekhab Alam et al. [9] developed experimental and theoretical studies on lead free perovskites, showing that CH_3_NH_3_SnI_3_ (MASnI_3_) has an optimal band gap of 1.3 eV, which covers a wide part of the visible solar spectrum. Piyush K. Patel [10] and Sagar Bhattarai et al. [11] performed simulations with perovskite CH_3_NH_3_SnI_3_ under AM 1.5G illumination; they studied the effect of thickness, acceptor concentration and defect density in the absorber layer, and their *PSCs* achieved a *PCE* of 28.39% and 22%, respectively. In addition, Teodor Todorov et al. [12] and Yousaf Hameed et al. [13] have worked with perovskite and kesterite in their solar cell structures, obtaining a *PCE* of 16% and 19.52%, respectively.

The most used organic materials such as HTM in *PSCs* are conductive polymers, like PTAA and Spiro-OMeTAD. Despite the remarkable performance of both materials, they are generally very expensive, and they usually need doping with lithium (Li) salt to in-crease their conductivity and holes mobility [14]. For this reason, the application of organic HTMs in *PSCs* can be complicated, and it is necessary to develop low cost HTMs with greater stability. Certainly, inorganic materials are an alternative that can be used for this purpose, and they have advantages in terms of synthesis, high stability and low cost.

Cu_2_ZnSnS_4_ (CZTS) is an inorganic material and quaternary semiconductor compound of group I-II-IV-VI which crystallizes in a structure that can be of the kesterite or stannite type [15]. Thin films of CZTS are composed of abundant materials on earth with low toxicity, and they have suitable properties for photovoltaic application. CZTS has a tunable direct band gap energy (in the range of 1.4–1.6 eV) and a large absorption coefficient (around 10^4^ cm^−1^) that make it an excellent and promising material for solar cells [14,16]. CZTS films can be synthesized by sputtering deposition technique, evaporation, spray pyrolysis and pulsed laser deposition (PLD) [17,18].

The effectiveness of CZTS as a low cost HTM has been competitive compared to Spiro-OMeTAD in *PSC*. Qiliang Wu et al. [14] have used a CZTS film as a hole conductor beyond the traditional light absorber, achieving a *PCE* of 12.75%. Xin Li et al. [19] successfully developed a solution-processed CZTS as HTL for inverted *PSCs*, the proposed structure was FTO/CZTS/Perovskite/PCBM/Ag, obtaining a *PCE* of 13.75%. Suzanne K. Wallace et al. [20] identified challenges and opportunities in CZTS solar cells such as a chemical treatment to activate the absorber layer, improvement on carrier generation, transport, recombination and collection, defect quantification and alternative architectures for solar cells. Ismaila Taiwo Bello et al. [21] performed solar cells simulations using perovskite sandwiched between kesterite layers with a planar heterojunction structure, achieving a *PCE* of 22.57%., proving that a structure combining perovskite and kesterite is a promising solar cell configuration.

Recently, the use of simulation software in the area of solar cell devices has been of great importance; one of the most widely used and which has contributed to scientific articles is SCAPS -1D. It is a one-dimensional solar cell simulation software developed at the department of electronics and information systems (EIS), University of Gent, Belgium [22].

SCAPS-ID has proven to be of vital importance to understand the behavior of solar cells under certain working conditions. Table 1 shows different solar cell structures simulated by SCAPS-1D; there is not optimal structure to date, and different research groups keep working to improve the efficiency of third generation solar cells.

In this work, we study and evaluate with SCAPS-1D, the electrical performance of a *n-i-p* planar solar cell structure consisting of FTO/TiO_2_/MASnI_3_/CZTS/Au. We proposed a lead-free perovskite MASnI_3_ as absorber layer and CZTS as HTL in the solar cell device to improve the *PCE*. These efficient charge transport layers play an important part in the performance of these devices because they assure charge transfer at the anode or cathode interface, therefore, guiding the charge carriers to electrode terminals. The simulations allowed the optimization of solar cell parameters such as: open circuit voltage (*V_oc_*), short circuit current (*J_sc_*), fill factor (*FF*) and *PCE*.

## 2. Materials and Methods

Simulation has become a very important tool for researchers because it allows a detailed understanding, and also, it contributes to experimental optimization and further evaluation to study the optical and electrical parameters of the materials and, in this way, predict the behavior of the devices.

Numerical Simulation SCAPS-1D is a numerical modelling tool aimed at simulation of the properties of semiconductor structures. The model is based on solving the basic semiconductor equations (the Poisson equation and the electron and hole continuity equations). In bulk form, the formulas are denoted below with the constitutive Equations (1)–(5) [23].

The Poisson equation shows the relationship between the electric field of a *p*−*n* junction (E) and the space charge density (ρ):

(1) $$\frac{\partial^{2}\psi }{\partial^{2}x}=-\frac{\partial E}{\partial x}=-\frac{\rho }{\varepsilon_{s}}=-\frac{q}{\varepsilon_{s}}\left[p-n+N_{D}^{+}\left(x\right)-N_{A}^{-}\left(x\right)\pm N_{def}\left(x\right)\right],$$

where

ψ electrostatic potential,

q elementary charge,

$\varepsilon_{s}$ static relative permittivity,

p, n electron and hole density,

$N_{D}^{+},\;N_{A}^{-}$ density of ionized donors and acceptors,

$N_{def}$ defect density (acceptor or donor).

The electron and hole continuity equations in steady state are

(2) $$\frac{\partial j_{n}}{\partial x}+G-U_{n}\left(n,p\right)=0,$$

(3) $$-\frac{\partial j_{p}}{\partial x}+G-U_{p}\left(n,p\right)=0,$$

where

$j_{n\left(p\right)}$ electron and hole current densities,

$U_{n\left(p\right)}$ recombination rates,

G electron–hole generation rate.

The electron and hole current density are expressed as:

(4) $$j_{n}=qn\mu_{n}E+qD_{n}\frac{\partial n}{\partial x},$$

(5) $$j_{p}=qn\mu_{p}E-qD_{p}\frac{\partial p}{\partial x},$$

where

q elementary charge,

$\mu_{n\left(p\right)}$ electron and hole mobility,

$D_{n\left(p\right)}$ diffusion coefficient of electrons and holes.

**Figure 1:**
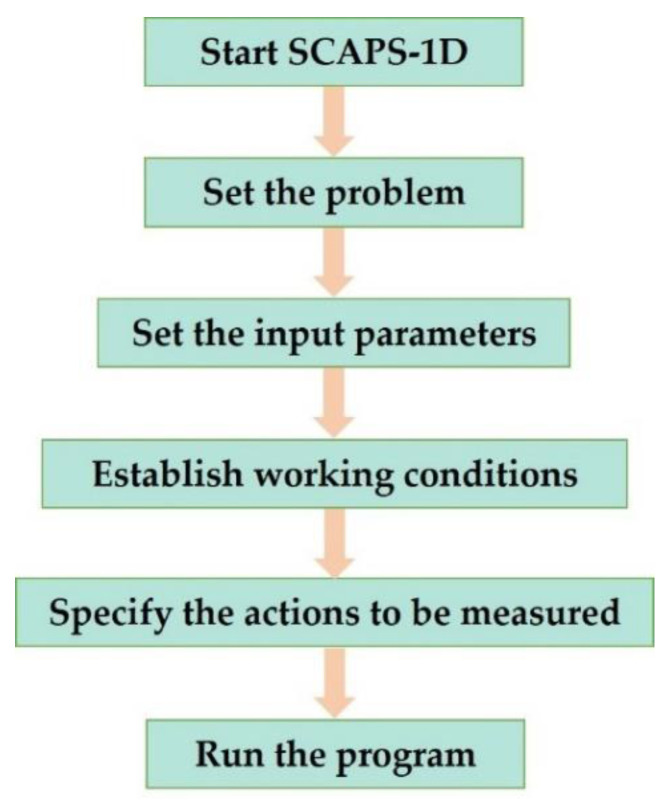
Steps to build and simulate a solar cell in SCAPS-1D.

Regarding to process simulation, the diagram in Figure 1 shown the steps to build and simulate the proposal device.

The proposed solar cell structure in this work consists of a Fluorine-doped tin oxide (FTO) substrate, an electron transporting layer (TiO_2_), a lead-free perovskite layer, a hole transporting layer (CZTS) and a gold back contact (Au).

**Figure 2:**
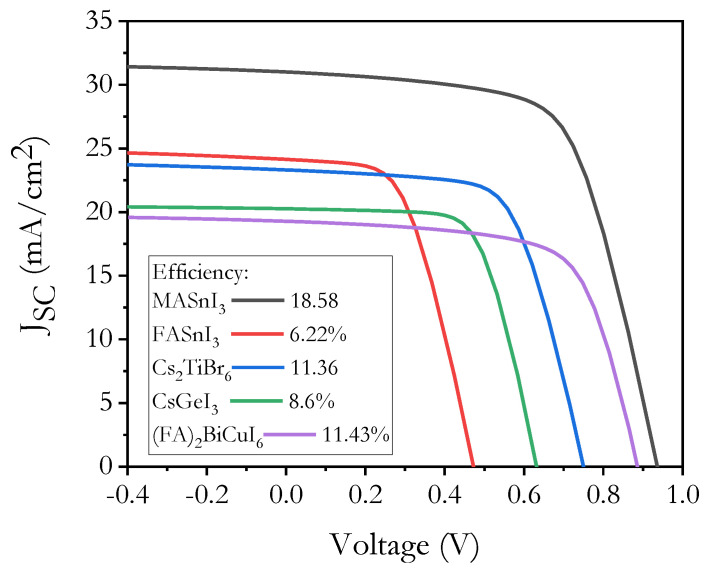
*J−V* characteristics of lead-free *n-i-p PSCs*.

Figure 2 shows the *J-V* characteristics of different FTO/TiO_2_/lead-free perovskite/CZTS/Au structures; the lead-free perovskites used in this graph were: MASnI_3_, FASnI_3_, Cs_2_TiBr_6_, (FA)_2_BiCuI_6_ and CsGeI_3_ [13,24,25,26,27].

These preliminary results showed higher values for *J_sc_*, *V_oc_* and *PCE* in the solar cell with a MASnI_3_ perovskite, and this supports the possibility to study and carry out the necessary optimization to achieve the best record efficiency for this pv device.

Table 1 shows the parameters *PCE*, *FF*, *V_oc_* and *J_sc_* reported in solar cells structures with perovskites (MASnI_3_ and NH_3_CH_3_PbI_3_) and kesterites (CZTS and CZTSSe) using SCAPS 1-D and experimental results. It can be appreciated that these devices were selected because they share a similar structure to the one proposed in this work.

**Figure 3:**
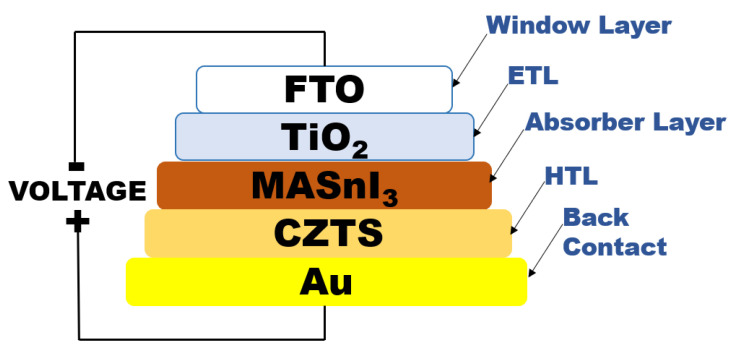
The *n-i-p* perovskite solar cell structure.

Figure 3 shows the structure of the perovskite solar cell proposed using SCAPS-1D. The device is composed of a *n-i-p* (*n*: electron transporting layer, *i*: intrinsic layer, *p*: hole transporting layer) planar heterojunction.

The structure of the solar cells is the following: transparent conductive oxide (TCO)/electron transporting layer (ETL)/absorbent layer (perovskite)/hole transport material (HTM)/back contact. According to the structure mentioned before, the materials used were SnO_2_:F (FTO)/TiO_2_/MASnI_3_/CZTS/Au.

It is a one-dimensional device with *n-i-p* planar heterojunction; the *n* region is the ETL; the *i*-region is the perovskite layer, and the *p*-region is the HTL. When a solar cell device is exposed to light, the diffusion length of excitons that come mainly from the perovskite layer allows to reach the *n* and *p*-regions. At the *n-i* interface, the exciton is dissociated, and the electron moves toward the *n*-layer while the hole migrates towards the *p*-layer. Similarly, at the *i-p* interface, the exciton is dissociated, and the hole moves to the *p*-layer while the electron migrates to the *n*-layer. The dissociation of exitons and the migration of electrons and holes is favored by the electrical field between the *n* and *p*-layers.

The optical and electrical parameters used in SCAPS-1D for the device model are shown in Table 2; they are the following: thickness of the absorbent layer, the electron transport layer and the hole transport layer; and their electron/hole mobilities, carrier doping concentrations, electron affinities, band gaps and doping densities.

In our planar solar cell configuration, the perovskite film is the absorber layer “sandwiched” between the HTL and ETL layers, CZTS and (TiO_2_), respectively. These charge transport layers play an important part on the performance of these devices. Efficient charge transport layers assure the charge transfer at anode or cathode interface, therefore, guiding the charge carriers to analogous electrode terminals. Generally, after illumination, the absorbent layer generates electron–hole pairs. The generated charge carriers then diffuse to an ETL (electrons) and HTL (holes) layer via interfaces, and the electrons get diffused into the conduction band (CB) of the ETL; furthermore, the holes are injected into the valence band (VB) of the HTL layer. To conclude, the holes and electrons are then collected by the conductive terminals, front contact and back contact, for our device (Au).

The simulation is carried out under an AM 1.5G solar spectrum with an incident power density of 1000 W/m^2^ at room temperature (300 K).

The main objective of this work is to demonstrate that the perovskite MASnI_3_ works perfectly with the CZTS and varying their thickness, band gap of CZTS, working temperature, acceptor density and defect density of perovskite to obtain the best efficiency.

In Table 3, the parameters of back and front contacts are shown. The gold (Au) metal is chosen as the back contact, due its good conductivity and high optical reflectivity to reflect photons back to the absorber layer, and the metal work functions are 5.1 eV and 4.4 eV for Au and FTO, respectively [24].

SCAPS-1D software gets the basic characteristics of the solar cell, such as the band diagram, generation and recombination rates, and cell current densities.

**Figure 4:**
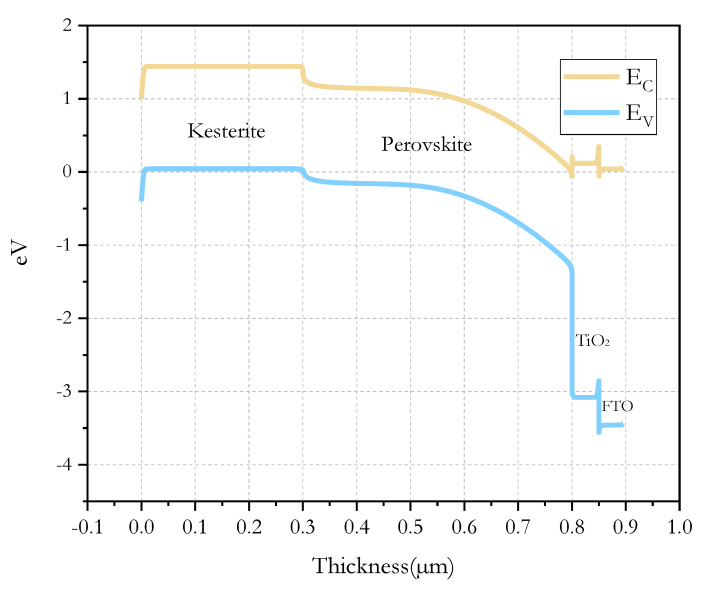
The band diagram of the *n−i−p PSC* at equilibrium.

The band diagram of the *n-i-p PSC* at equilibrium is shown in Figure 4. It can be seen that perovskite and kesterite used in the solar cell simulation present a similar band gap of 1.3 and 1.4 eV, respectively. The position of the conduction band and the valence band allows affinity between perovskite and kesterite, so there is no barrier that blocks the charge carriers, and thus, the band alignment is one of the most important parameters that influence the current transport across the heterojunction and the performance of the solar cells.

**Figure 5:**
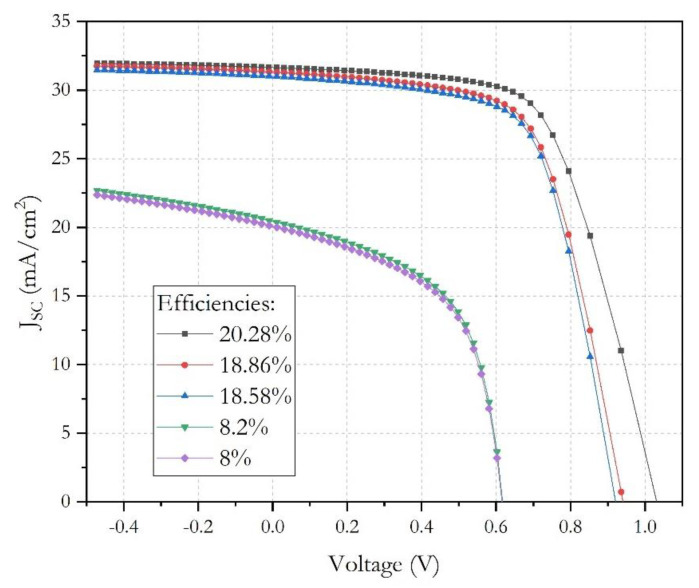
*J−V* characteristics of the *n-i-p PSC*.

The curves of current density–voltage (*J−V*) characteristic and quantum efficiency–wavelength (*QE-λ*) were obtained, and these are shown in Figure 4 and Figure 5, respectively. Under AM1.5G light conditions, the solar cell produces hole–electron pairs, and the current begins to flow, due to charge carriers generated by the incidence photons. The obtained *J-V* characteristics and the extracted output parameters are summarized and showed in Figure 5 and Table 4.

In Table 4, a summary of main electrical parameters is showed; when physical parameters in perovskite and kesterite were changed, the parameters that remained constant were not included as indicated in Table 2. First simulations showed a low *PCE* value of 8.00% when the highest value of 2 × 10^15^ for *N_t_* and the lowest value of 10^16^ for *N_A_* were used; these values confirm the importance on the absorbent perovskite quality and the influence on the interface passivation [33]. *PCE* was clearly increased to 18.58% as a result of decreasing the loss mechanisms on interface when *N_t_* was reduced to 2 × 10^14^ [34]. Finally, the best *PCE* value of 20.28% was obtained when *N_A_* was increased up to 1 × 10^19^ and kesterite thickness was 300 nm, as a consequence on the increase of absorption properties in perovskite and kesterite layers that will be discussed in the following sections.

The measure of a photovoltaic cell quality is the fill factor (*FF*). The *FF* is calculated by equating the maximum power (*P_max_*) to the theoretical power (*P_t_*) that would be output at both the short circuit current (*J_sc_*) and open circuit voltage (*V_oc_*) together as given in Equation (6). The ratio of the energy output from the photovoltaic solar cell to the energy input from the sun is the power conversion efficiency (*PCE*) and is mathematically expressed in Equation (7) [35].

(6) $$FF=\frac{P_{max}}{P_{t}}=\frac{V_{max}I_{max}}{V_{oc}J_{sc}},$$

(7) $$PCE=\frac{V_{oc}J_{sc}FF}{P_{in}}.$$

**Figure 6:**
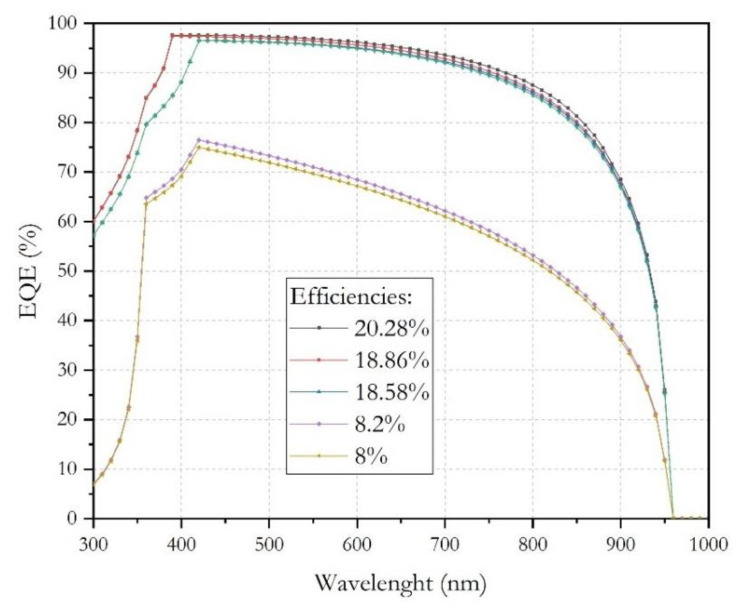
The external quantum efficiency of the *PSC*.

In Figure 6 are shown all external quantum efficiency (*EQE*) simulation, can be appreciated an excellent *EQE* > 90%, where we cover the entire range of the visible spectrum and is related to a good current density *J_sc_* = 31.66 mA/cm^2^.

Solar cells have high series resistance (*R_s_*), and in this study, the *R_s_* values were varied to see the effect of variation in efficiency; in this way, it was decided to use 5 Ω-cm^2^, to obtain a result more adjusted to the solar cell real operation [36].

As mentioned, in Nandi Wu et al. [37], one of the parameters in a solar cell that is most difficult to optimize is the *FF*, since it is sensitive to parasitic loss mechanisms and generally has a lesser impact on *V_oc_* and *I_sc._*

It is known that the presence of defects in the absorbent layer limits the *FF* below the theoretical achievable value.

Some mechanisms that cause the loss of *FF* in the performance of the solar cell are misalignment of the conduction band (CB) between ETL and the absorber layer and misalignment of VB between HTL and the absorber layer, which is not our case. However, another cause of *FF* reduction is mainly series resistance, and a significant fraction is contributed by transport resistance within the absorber.

As mentioned above, series and shunt resistance can cause even greater reduction in *FF* in solar cell devices. In a simple solar cell heterojunction model, these resistances are ohmic elements whose presence can be detected with specific changes in the *I-V* curve.

A suitable fill factor *FF* was obtained with CZTS as HTL, and it could be related to the low series resistance (*R_s_*) 5 Ω-cm^2^. A doping density of 10^19^ cm^−3^ of CZTS led to a reduced depletion region in the HTL of the device, which is a thin layer with 300 nm of thickness. This phenomenon reduces the bulk resistance of the *p*-type material and the interfacial contact resistance between back contact and the *p*-type layer.

## 3. Analysis of Absorber Layer Thickness

The photovoltaic parameters *J_sc_*, *V_oc_*, *FF* and *PCE* are strongly influenced by the thickness of the absorbent layer (perovskite). To obtain the optimum value of the absorbent layer in the simulation, the thickness was varied from 250 to 1000 nm.

**Figure 7:**
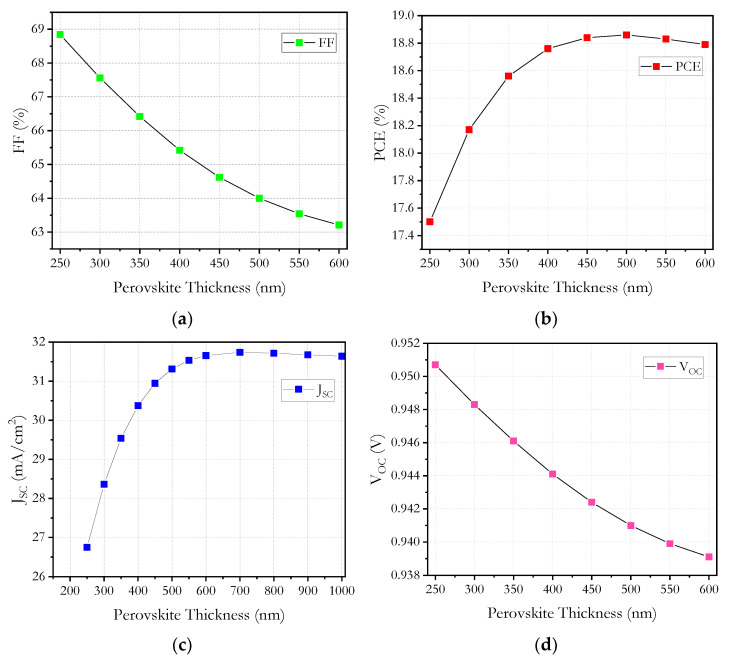
Absorber layer thickness versus (**a**) *FF*, (**b**) *PCE*, (**c**) *J_sc_* and (**d**) *V_oc_*.

In the results in Figure 7, it is observed that *J_sc_* increases steeply up to 500 nm and then varies slowly with thickness. The best *J_sc_* value was obtained at 31.74 mA/cm^2^ with an optimal thickness of 700 nm and then decreased slightly; this is mainly attributed to the large absorption coefficient.

The *V_oc_* had a small decrease of 0.013 volts, which can be attributed to the improved recombination of the free charge carriers in the thicker absorbent.

Similar behavior is shown in the *FF*, decreasing with increasing thickness, and this effect may be due to the increase in series resistance (*R_s_*). The *FF* is an important parameter to evaluate the performance of solar cells and is considered an ability to deliver available power to a load generated by a cell; therefore, it describes the internal power depletion. In thicker absorbers, the internal power depletion enhances and causes a reduction of *FF*.

The *PCE* reaches a maximum value of 18.86% at 500 nm, and this parameter decreases with a further increase in the thickness of the absorbent. This behavior is due to the fact that the thickness of the absorber is less than the diffusion length of the charge carriers; therefore, most of the charge carriers reach the electrodes and thereby increase the *PCE*. On the other hand, recombination occurs in a thick absorbent layer, thereby causing *PCE* to decrease with a further increase in thickness.

## 4. Analysis of Hole Transporting Layer Thickness

Spiro-OMeTAD has been used mostly as a HTL in perovskite solar cells. However, the pristine Spiro-OMeTAD has a low hole mobility and a low acceptor concentration; the series resistance at open-circuit is thus limited by insufficient hole-conduction [38].

As the conductivity of pristine spiro-OMeTAD is too low to achieve high performance in perovskite solar cells, researchers have found necessary to dope this material to improve conductivity and avoid charge recombination at the spiro–OMeTAD/perovskite interface.

Some techniques have been developed to improve the conductivity and extraction holes of Spiro-OMeTAD, and this has been achieved by doping this material with benzoyl peroxide (BPO) which is commercially available. In this way, the load transfer and therefore the energy conversion efficiency can be improved [39].

[In_0.5_K (3-qlc) Cl_1.5_ (H_2_O) _0.5_] _2n_ (shorted as In10) have been successfully incorporated into Spiro-OMeTAD, thus achieving oxidation to Spiro-OMeTAD^+^, and in this way, the conductivity was increased, improving the charge transport and suppressing charge recombination in *PSC* [40].

Taking into account what was mentioned above, the relevance of this work is based on the use of CZTS, instead of Spiro-OMeTAD, which is quite expensive. It has the advantage of having similar band gaps, 1.3 eV for MASnI_3_ and 1.4 eV for CZTS, and an excellent affinity in their interface.

**Figure 8:**
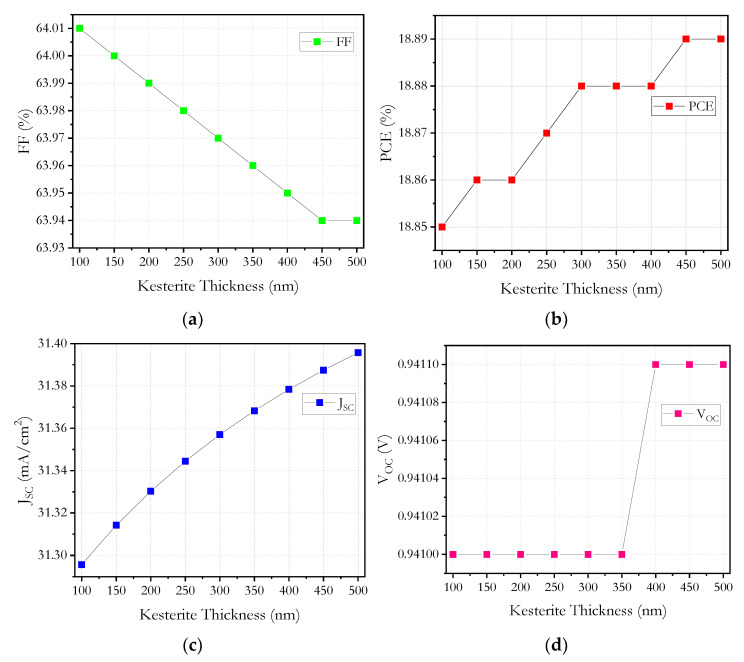
Kesterite layer thickness versus: (**a**) *FF*, (**b**) *PCE*, (**c**) *J_sc_* and (**d**) *V_oc_*.

It could be thought that if thickness is increased, then more photons are absorbed, and as a result, more electron hole pairs are generated, but the simulation shows that there is not important change in the *PCE*, *FF, J_sc_* and *V_oc_* values with the change in CZTS thickness; see Figure 8.

When the thickness of the kesterite increases above 300 nm up to 500 nm, an increase of 0.01% for the *PCE* (Figure 8b), a decrease of 0.03% for the *FF* (Figure 8a), an increase of 0.04 mA/cm^2^ for the *J_sc_* and an increase of 0.0001 Volts for the *V_oc_* can be observed. In order to show the small changes in *FF* and *J_sc_*, an enlarged scale was used for the *Y*-axis in Figure 8b,c, and it is possible to observe that there are no significant changes.

Given the behavior of kesterite, it was decided that a value above 300 nm does not provide an improvement in the solar cell parameters. A greater thickness of kesterite does not increase efficiency since there would be greater travel in the charge carriers, and it would have a greater probability of recombination, in addition to the series resistances growing and internal power depletion causing a reduction in the *FF*.

The use of a CZTS layer allows the *EQE* curve to have a greater amplitude, reaching up to 950 nm; despite the fact that in other structures the hole-transporting layer used works as an absorber layer, in our case, we cannot affirm that it helps to the perovskite in the absorption of photons. In this way the highest absorption is attributed to perovskite.

## 5. Tuning Band Gap Energy in Kesterite Layer (HTL)

Pure Spiro-OMeTAD material is the most used today for solar cells as HTL; it has low hole mobility and low acceptor density, which cause a high series resistance (*R_s_*), restricting the current in the device and leading to an undesirable performance [41].

One promising absorber material is the CZTS due to its high absorption coefficient of over 10^4^ cm^−1^ and direct and optimal band gap of about 1.4 eV [14,16], coupled with the fact that its physical properties can be tailored.

However, the record efficiency of about 10% obtained for a single junction CZTS solar cell is still very low when compared to its theoretical efficiency of 28% [42]. The excellent properties of Cu_2_ZnSnS_4_ (CZTS) quaternary compound makes it as promising a material as the absorber layer for thin film solar cells (*TFSCs*).

**Figure 9:**
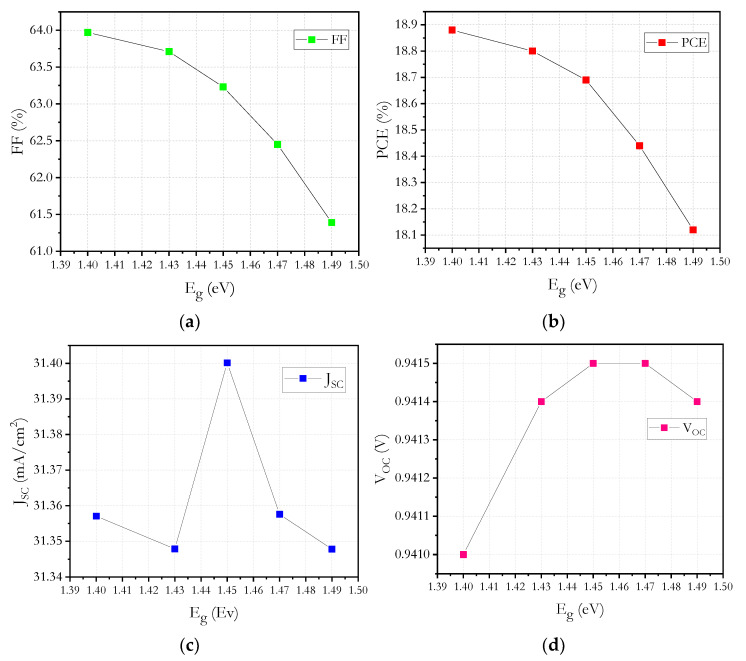
Kesterite band gap energy versus: (**a**) *FF*, (**b**) *PCE*, (**c**) *J_sc_* and (**d**) *V_oc_*.

In our simulation work, the influence of energy band gap of CZTS on cell performance is also investigated. We kept unchanged the thickness of CZTS and varied the band gap from 1.4 to 1.49 eV, and simulation results are shown in Figure 9. It can be seen that by increasing the band gap of CZTS, *V_oc_* and *J_sc_* remain constant with 0.94 V and 31.3 mA/cm^2^, respectively.

Some relevant changes were observed in the *PCE* and the *FF*, in which the best value is presented in 18.88% and 64%, respectively.

## 6. Analysis of Defect Density (*N_t_*) in Absorber Layer

The defects density *N_t_* is a very important parameter that affects the performance of the solar cell. Defect density is based on the Shockley–Read–Hall model [4]. The model is given in Equation (8):

(8) $$R=\frac{np-n_{i}{}^{2}}{\tau_{p}\left(n+N_{C^{e}}{}^{\left(E_{g}-E_{t}\right)/kT}\right)+\tau_{n}\left(p+N_{v^{e}}{}^{\left(E_{t}\right)/kT}\right)},$$

where *n* and *p* represent the concentration of electrons and holes, which are obtained by the solution of continuity and Poisson equations; *n_i_^2^* term is neglected because *qV* > *3kT* for adequate forward bias.

There is a reason why a solar cell cannot achieve maximum theoretical conversion efficiency; due to the dependence of this parameter on the defect density *N_t_* of the absorbent layer, the defects cause a negative effect on the diffusion length of the photogenerated carriers and therefore affect the useful life of the device.

The aforementioned effect is expressed in Equations (9) and (10); in Equation (9), the relationship between diffusion length, mobility and useful life of the device is shown [43].

(9) $$L_{D}=\sqrt{\frac{kT\mu_{\left(e,h\right)}}{q}\tau_{lifetime}},$$

*L_D_* is the diffusion length, *µ*_(*h,e*)_ are the electron and hole mobility, and *τ_lifetime_* is the minority-carrier lifetime. *τ_lifetime_* depends of defect trap density and capture cross-section area for electrons and holes. The relation between *τ_lifetime_* and the bulk defect density is showed in Equation (10) [43].

(10) $$\tau_{lifetime=\frac{1}{N_{t}\delta v_{th}}},$$

where *δ* represents the capture cross-section area for electrons and holes, $v_{th\;}$ represents the thermal velocity of carriers, and *N_t_* is traps density.

From the previous equations, we can see that increasing traps density, the diffusion length of the carrier will be limited, thus causing a lower probability of carriers reaching contact.

The diffusion length can be affecting the performance of the solar cell and limit the thickness of the absorbent layer.

Referring to the results shown in the Figure 6, it can be concluded that the optimal thickness of the absorber layer will be limited by the minority carrier–diffusion length and density of defects in the absorber layer.

**Figure 10:**
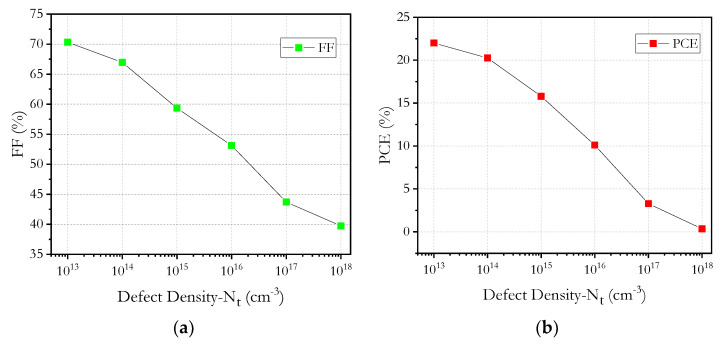

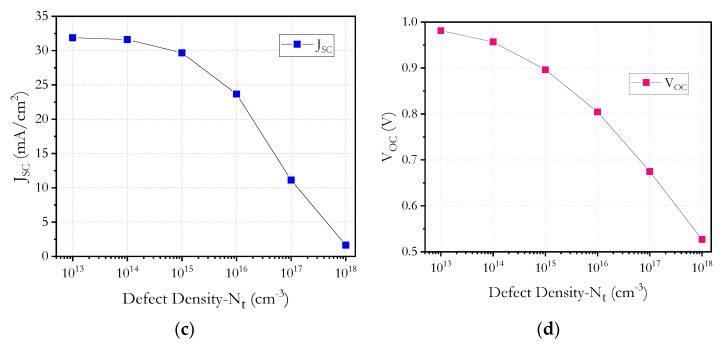
Absorber layer defects *N_t_* versus: (**a**) *FF*, (**b**) *PCE*, (**c**) *J_sc_* and (**d**) *V_oc_*.

The graphs in Figure 10 show that defect density (*N_t_*) of the absorber layer varied between 10^13^ cm^−3^ to 10^18^ cm^−3^. With the increase of *N_t_* of the absorbent layer, the *PCE*, *FF*, *V_oc_* and *J_sc_* of the solar cell decreased remarkably. However, by decreasing *N_t_*, it is observed that all the cell parameters greatly improve; 10^14^ cm^−3^ was taken as the best density of defects of the absorbent layer. The best absorber defect density was the same as in the reviewed literature.

## 7. Analysis of Acceptor Carrier Concentration (*N_A_*) in Absorber Layer

**Figure 11:**
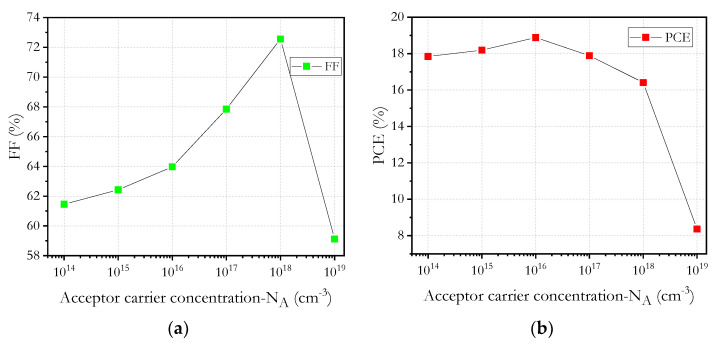

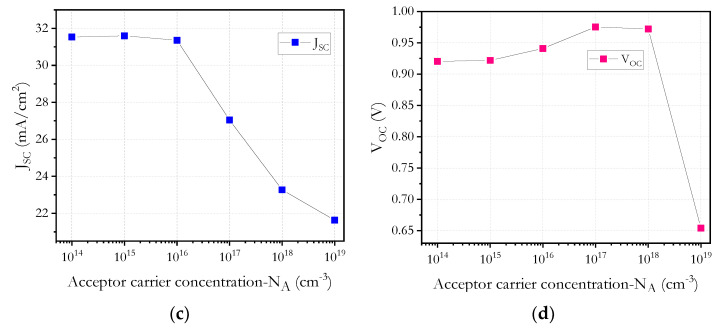
Acceptor carrier concentration (*N_A_*) in absorber layer versus: (**a**) *FF*, (**b**) *PCE*, (**c**) *J_sc_* and (**d**) *V_oc_*.

Results were obtained by varying acceptor carrier concentration *N_A_* of the active tin perovskite layer. The Figure 11 shows the behavior of the cell parameters when the acceptor density in the absorbent layer is modified.

When the *N_A_* increases from 10^14^ to 10^19^ cm^−3^, small increases in *FF* are perceived until reaching 10^18^ cm^−3^, from that point on, a notable drop begins to be observed; this decrease is due to the fact that when the doping concentration increases, mobility increases, and the useful life decreases, which translates into a decrease in the duration of diffusion.

When we increase the acceptor carrier concentration, the Fermi energy level of holes decreases and, consequently, a slight increase in *V_oc_* can be observed, as shown in Figure 11. Another effect is that the potential increases with the increasing acceptor carrier concentration; due to this fact, charge separation is promoted and therefore *V_oc_* is increased. However, *J_sc_* initially increases slightly to 31.6 mA/cm^2^ at 10^15^ cm^−3^ and then falls dramatically to 21.63 mA/cm^2^ at 10^19^ cm^−3^. This effect can be attributed to the increased recombination rate of carriers of loading within the absorbent layer of perovskite.

However, when *N_A_* exceeds 10^16^ cm^−3^, a drop in *PCE* is observed. The defective state of the absorbent layer leads to a considerable drop in energy conversion efficiency, as shown in Figure 11. The behavior of the *PCE* solar cell is convincing, and the best result of 18.9% is obtained with 10^16^ cm^−3^ acceptor carrier concentration.

## 8. Effect of Operating Temperature

The operating temperature has a main impact on the performance of a solar cell. Normally, the standard operating temperature for a solar cell is taken to be 300 K; nevertheless, during the operation the cell is subjected to an even higher temperature.

The *PCE*, *V_oc_*, *J_sc_* and *FF* of the solar cell decreased because the carrier concentrations, mobility of the charge carriers, resistance and bandgap of the materials alter at a higher temperature [9].

**Figure 12:**
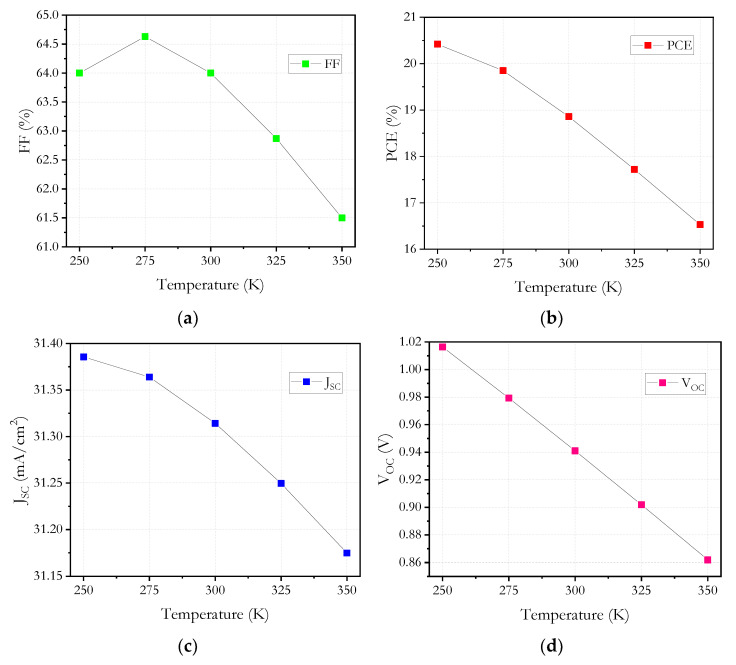
Effect of working temperature versus: (**a**) *FF*, (**b**) *PCE*, (**c**) *J_sc_* and (**d**) *V_oc_*.

For the analysis of the proposed solar cell, the operating temperature was varied from 250 K to 350 K. The transport layers are responsible for collection of the charge carriers towards the charge collective contact. The various materials exhibit variable behavior in temperature fluctuations because all vary in conductivity, specific heat and density [42]. The results of the effect of temperature on *V_oc_*, *FF*, *J_sc_* and *PCE* are represented in Figure 12. With increasing temperature, the loss of *V_oc_* is greater; those phenomena occur similarly for *FF*, *J_sc_* and *PCE*.

To understand how temperature influences a solar cell, the following Equation (11) is presented [44].

(11) $$V_{oc}=\frac{E_{A}}{q}-\frac{nKT}{q}ln\left(\frac{J_{00}}{J_{sc}}\right),$$

where *V_oc_* is open circuit voltage, *E_A_* is activation energy, *n* is diode ideality factor, *K* is constant of Boltzmann, *T* is temperature in Kelvin, *J*_00_ is current prefactor, and *J_sc_* is short circuit current.

From Equation (11) it can be seen that with increasing temperature, *V_oc_* will decrease significantly. This further suggests that the increase in reverse saturation current is the reason behind decrement of *V_oc_* with an increase in temperature.

The temperature is an important factor, because when the ambient temperature increases, the output power is lower in a photovoltaic module. In other words, a solar panel works more efficiently when is subjected to a lower temperature. In this way, the solar cell converts a greater proportion of the solar radiation it receives into electricity.

Analyzing the results obtained in the literature for the architecture of a simple perovskite/kesterite heterojunction, it was possible to improve the efficiency of the reported solar cell by 4%, obtaining with our efforts in simulation an efficiency (*PCE*) of 20.28%.

Certainly, the simulation study can be generalized if it is the same structure, since if it is a different structure, the optimal and electrical parameters, the transport mechanisms and the physics of the device would change. Therefore, the analysis of the results obtained will allow to know how the solar cell will behave and, in this form, improve the performance of the device and offer an approach to what is possible to obtain in the laboratory under certain working conditions.

## 9. Conclusions

In this work, a lead-free perovskite MASnI_3_ solar cell with structure FTO/TiO_2_/CH_3_NH_3_SnI_3_/CZTS/Au was simulated using SCAPS 1-D.

The photovoltaic parameters were optimized by varying the thickness of the absorber layer (perovskite) and HTL (kesterite), the CZTS band gap, the acceptor carrier concentration *N_A_*, defect density *N_t_* and the working temperature of the device.

The thickness of perovskite MASnI_3_ layer and kesterite CZTS layer were optimized to 500 nm and 300 nm, to improve the *PCE*.

For the absorber layer, the ideal *N_A_* and *N_t_* were 10^16^ cm^−3^ and 10^14^ cm^−3^, respectively; the band gap of kesterite CZTS was optimized to 1.4 eV, and a series resistance (*R_s_*) of 5 Ohm-cm^2^ was used.

Finally, it can be observed that the increase in the working temperature has a negative effect on the parameters of the solar cell, so despite obtaining better results at lower temperatures, it was decided to a temperature of 300 K would produce more representative results.

Results indicate that proper defect density improves cell performance; however, excessive concentration leads to a higher recombination rate of charge carriers and poor cell performance.

The Schottky junction was formed at an anode Au/CZTS interface since Au has a high metal work function that is required for the ohmic contact.

The final parameters that were achieved after optimization of the solar cell are *J_sc_* = 31.66 mA/cm^2^, *V_oc_* = 0.96 V, *FF* = 67% and *PCE* = 20.28%. This last result (*PCE*) is 4% better than that reported and reviewed in the literature for this solar cell structure.

The reported *PSC* using MASnI_3_ provides a viable path to achieving a *PSC* that does not have the toxicity of lead, as well as low cost and high efficiency that could be applied in the field of energy harvesting systems.

## Figures and Tables

**Table 1:** Parameters *V_oc_*, *J_sc_*, *FF* and *PCE* reported in solar cells using SCAPS-1D simulation and experimental results.

Solar Cell Structure	*V_o_*_c_ (V)	*J_sc_* (mA/cm^2^)	*FF* (%)	*PCE* (%)	Ref.
o/Cu_2_ZnSn(S,Se)_4_/CdS/ITO/PEDOT:PSS/CH_3_NH_3_PbI_3_/PCBM/Al	1.40	15.50	73.60	16.00	[12]
FTO/TiO_2_/MASnI_3_/CZTSe	0.86	29.45	76.74	19.52	[13]
FTO/TiO2/CH3NH3SnI3/CZTS/Au	1.06	20.54	58.70	12.75	[14]
FTO/TiO_2_/CH_3_NH_3_SnI_3_/CZTS/Au	0.94	20.31	72.00	13.75	[19]
ITO/CZST/CH_3_NH_3_PbI_3_/PCBM/BCP/Ag	0.82	9.70	76.10	6.02	[28]
ITO/NiO/meso-CZTSe: MAPbI_3_/ZnO/Al	1.05	19.19	83.30	16.80	[29]
FTO/TiO_2_/CH_3_NH_3_PbI_3_/CZTS/Ag	0.94	18.75	60.50	10.72	[30]
ITO/LT-CZTS/Perovskite/PCBM/PrCMA/Ag	0.92	20.70	81.00	15.40	[31]
FTO/TiO_2_/ZnO/CH_3_NH_3_Pb_0.5_Sn_0.5_I_3__−y_Cl_y_/CZTS/Pt-FTO	0.79	19.10	64.00	9.66	[32]
FTO/TiO_2_/MASnI_3_/CZTS/Au (this work [T.W.])	0.96	31.66	67.00	20.28	[T.W.]

**Table 2:** Perovskite solar cell input optical and electric parameters [13].

Parameters	Symbol	p-CZTS	i-MASnI_3_	n-TiO_2_	n-FTO
Thickness	W (nm)	300	500	50	50
Band gap	E_g_ (eV)	1.4	1.3	3.2	3.5
Electron affinity	X (eV)	4.1	4.17	3.9	4.3
Dielec. Permittivity	Ԑ_r_	9	8.2	9	9
(Conduction Band) Effective density of states	NC (cm^−3^)	2.20 × 10^18^	2.80 × 10^18^	1.00 × 10^21^	1.00 × 10^19^
(Valence Band) Effective	NV (cm^−3^)	1.80 × 10^18^	3.90 × 10^18^	2.00 × 10^20^	1.00 × 10^19^
Electron mobility	µ_e_ (cm^2^/Vs)	1.00 × 10^2^	1.60 × 10^−1^	2.50 × 10	2.50 × 10
Hole mobility	µ_p_ (cm^2^/Vs	1.25 × 10	1.60 × 10^−1^	1.00 × 10^2^	1.00 × 10^2^
Shallow uniform donor	n (1/cm^3^)	/	/	1.00 × 10^19^	2.00 × 10^18^
Shallow uniform acceptor	p (1/cm^3^)	1.00 × 10^19^	1.00 × 10^16^	/	/

**Table 3:** Back and front contact parameters.

Parameters	Symbol	Au	FTO
Metal work function	*ϕ* (eV)	5.1	4.4

**Table 4:** History of parameters of the simulations obtained in the solar cells.

*V_oc_* (V)	*J_sc_* (mA/cm^2^)	*FF* (%)	*PCE* (%)	Perovskite *N_A_*	Perovskite *N_t_*	Kesterite (nm)	TiO_2_ (eV)	TiO_2_ *N_D_*
0.77	20.45	51	8.00	1.00 × 10^16^	2.00 × 10^15^	100	3	9.00 × 10^16^
0.77	20.78	51	8.20	1.00 × 10^16^	2.00 × 10^15^	100	3	1.00 × 10^18^
0.94	31.00	64	18.58	1.00 × 10^16^	2.00 × 10^14^	100	3	1.00 × 10^19^
0.94	31.31	64	18.86	1.00 × 10^19^	1.00 × 10^14^	150	3.2	1.00 × 10^19^
0.96	31.66	67	20.28	1.00 × 10^19^	1.00 × 10^14^	300	3.2	1.00 × 10^19^
